# Supplementary material for: Real-time Alerting System for COVID-19 Using Wearable Data
Source: medRxiv. 2021 Jun 21:2021.06.13.21258795. Preprint. [Version 1] doi: 10.1101/2021.06.13.21258795 (PMC8240687; doi:10.1101/2021.06.13.21258795)

medRxiv preprint doi: https://doi.org/10.1101/2021.06.13.21258795; this version posted June 21, 2021. The copyright holder for this preprint (which was not certified by peer review) is the author/funder, who has granted medRxiv a license to display the preprint in perpetuity.
It is made available under a CC-BY 4.0 International license .

Supplementary figure and table legends:

Table1. Cohort. Demographics, health characteristics, and COVID-19 test and vaccination info of the Cohort.

Figure S1. Wearable devices distribution. 2,112 of participants had a smartwatch: 1,016 wore Fitbits, 950 wore Apple Watches, 93 wore Garmin, and 53 had other devices. Note that we

medRxiv preprint doi: https://doi.org/10.1101/2021.06.13.21258795; this version posted June 21, 2021. The copyright holder for this preprint (which was not certified by peer review) is the author/funder, who has granted medRxiv a license to display the preprint in perpetuity.
It is made available under a CC-BY 4.0 International license .

consider the device with the most amount of data as the main device in case of having more than one device.

Figure S2. Deterministic Finite State Machine in NightSignal algorithm. The state machine consists of six states, each labeled with an alert color and three symbols for transition between states based on the current average RHR overnight and the deviation level from the baseline (streaming median of averages of RHR overnight). For example, a red alert gets triggered if for two consecutive nights, the average RHR overnight is at least four bpm above the calculated baseline.

Figure S3. Comparing distribution of RHR overnight data in Fitbit vs. Apple Watch. To show the distribution of RHR data in Fitbit and Apple Watch, RHR overnight data points have been divided into 16 ranges (1-10, 10-20, etc) and each bar depicts the total number of nights that falls into each group. Each participant is presented with a color. Note that for the majority of participants, for most of the nights, there are 300 to 500 RHR overnight data points (i.e., almost minute resolution) in the case of Fitbit. However the range differs considerably in Apple Watch; the reason is that Apple Watch takes heart rate and step counts readings with different resolutions based on the activities. Note that in the NightSignal algorithm, we do not set a threshold for the minimum amount of data points required to aggregate per night. The first reason is that in most cases, even a very few data points are sufficient to get a proper average RHR overnight since we only consider HR records where the corresponding time interval (e.g. few minutes) for step count is zero. The second reason for that is if we do so (e.g., set the threshold to 40 data points), we will miss a significant amount of nights (e.g., first four bars in Fig. S3).

Figure S4. Thresholds and parameters in the NightSignal algorithm. As we discussed previously, the NightSignal algorithm uses the streaming median of average RHR overnight as

medRxiv preprint doi: https://doi.org/10.1101/2021.06.13.21258795; this version posted June 21, 2021. The copyright holder for this preprint (which was not certified by peer review) is the author/funder, who has granted medRxiv a license to display the preprint in perpetuity.
It is made available under a CC-BY 4.0 International license .

the baseline. We believe that it is a proper individual healthy baseline as we show that the fluctuation is insignificant and it usually deviates due to a long-term abnormal event (e.g., infection, medication consumption, vaccination). Fig. S4A shows the minimum number of nights required to hit a baseline close to the baseline over three months (within ± two bpm from the baseline - the reason behind choosing the threshold of two is that the baseline would still remain in the green zone) for the majority of participants. As depicted in the figure, for over 80% of participants, the proper baseline was observed after only seven nights. Fig. S4B depicts the range (max-min) of medians of average RHR overnight during three months of data with the median value of only three bpm. Similarly, Quer et al.[22] studied the variability in individual resting heart rate and showed that most subjects had a median weekly fluctuation in RHR of only three bpm. Given the fact that we only consider RHR overnight, the median fluctuation in RHR overnight is still three bpm even for a duration of three months. Similarly, Fig. S4C shows the corresponding standard deviation that is very low (0.8).

Figure S5. More examples of alerts for COVID-19 positive, COVID-19 negative, and potentially healthy participants. Shown are (from top to bottom): Signals for a COVID-19 positive case with mild and short-lasting symptoms. Note that this participant received zero red alerts during a healthy period of seven months and only received two red alerts starting the following night of symptoms onset date (the only period where the RHR overnight is much higher than baseline for three consecutive nights); an example for a sick participant with moderate symptoms followed by a COVID-19 negative test. Note that RHR overnight period began to increase three nights before the symptoms developed; the last two plots are the examples of healthy participants who reported no illness or symptoms of any kind during the study (except some poor sleep points for P174112). Both participants received no red alerts for over a three months healthy period (Note that for P174112, alcohol consumption affects the RHR overnight but the impact is either only for one night or not severe enough to trigger a red alert).

medRxiv preprint doi: https://doi.org/10.1101/2021.06.13.21258795; this version posted June 21, 2021. The copyright holder for this preprint (which was not certified by peer review) is the author/funder, who has granted medRxiv a license to display the preprint in perpetuity.
It is made available under a CC-BY 4.0 International license .

Figure S6. Impact of non-infectious events on NightSignal alerts (all day vs. overnight). Examples of the impact of different events (e.g., home and work stress, travel, and intense exercise) on the alerts based on two configurations: all day vs. overnight. For each participant, comparing the plots shows that the NightSignal algorithm reduces possible false positives due to non-infectious events by analyzing RHR overnight.

Figure S7. Impact of winter holidays on NightSignal alerts. As shown previously, there is a noticeable increase in the number of alerts during winter holidays -- particularly late December and beginning of January -- (“holiday bump”) due to the higher rate of travel, alcohol, entertainment, stress, and illness compared to other times of the year.

Figure S1: Wearable Devices Distribution

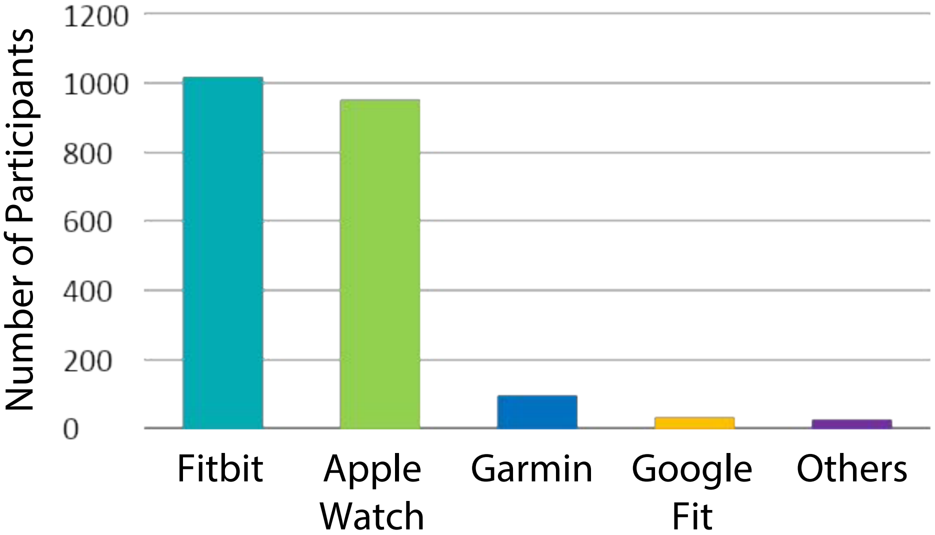

Figure S2: Deterministic Finite State Machine in NightSignal algorithm

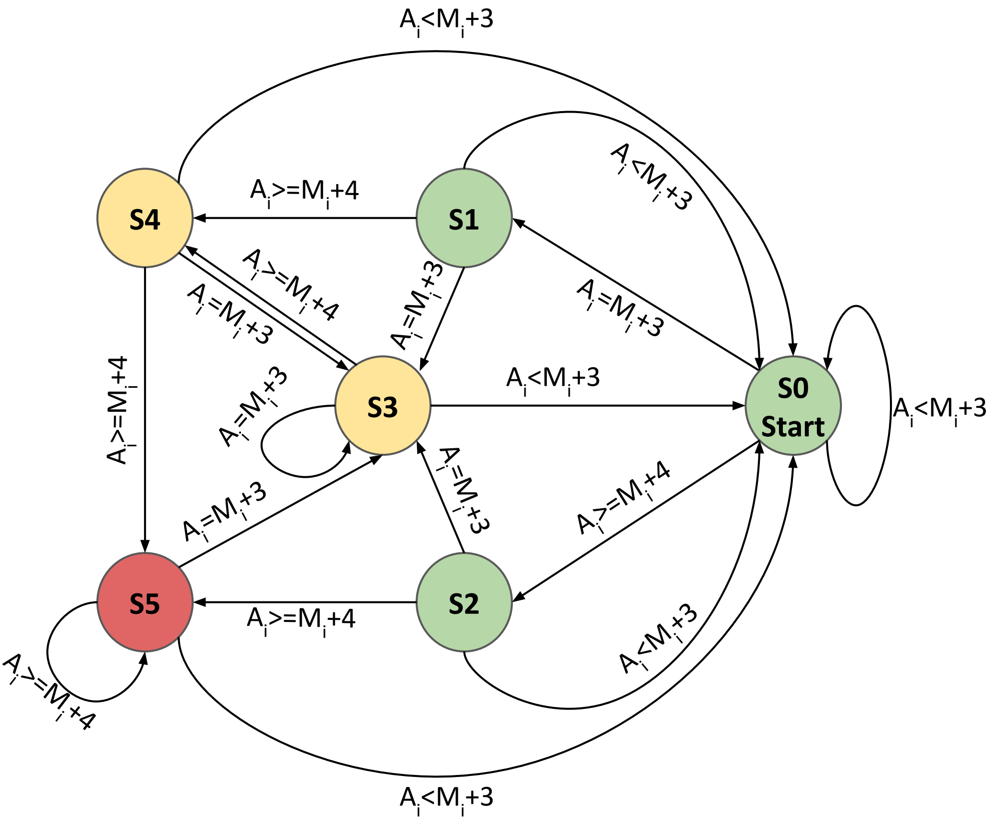

A$_i$: Average resting heart rate overnight for night *i*
M$_i$: Median of averages of resting heart rate overnight for all nights upto night *i*

Figure S3: Distribution of RHR Overnight Data in Fitbit vs. Apple Watch

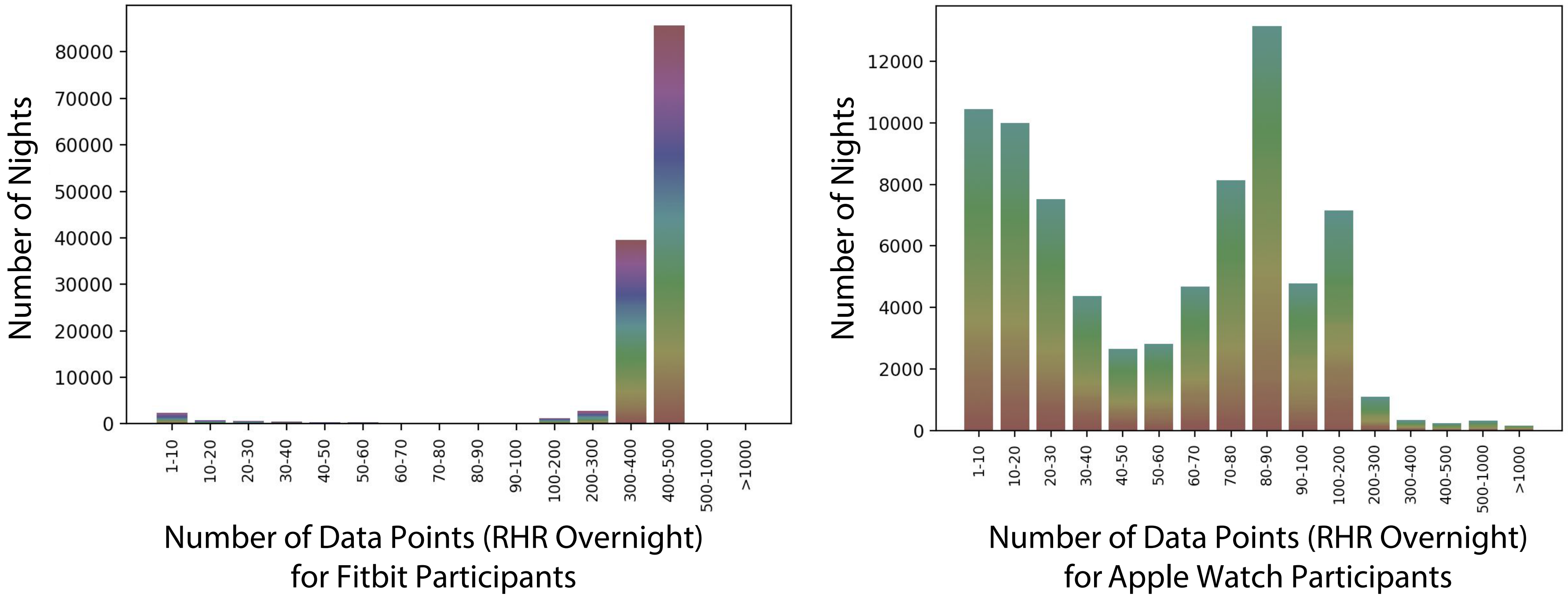

Figure S4: NightSignal Baseline Parameters and Fluctuation of Medians of Average RHR Overnight for Three Months

medRxiv preprint doi: https://doi.org/10.1101/2021.06.13.21258795; this version posted June 21, 2021. The copyright holder fo
(which was not certified by peer review) is the author/funder, who has granted medRxiv a license to display the preprint in
It is made available under a CC-BY 4.0 International license .

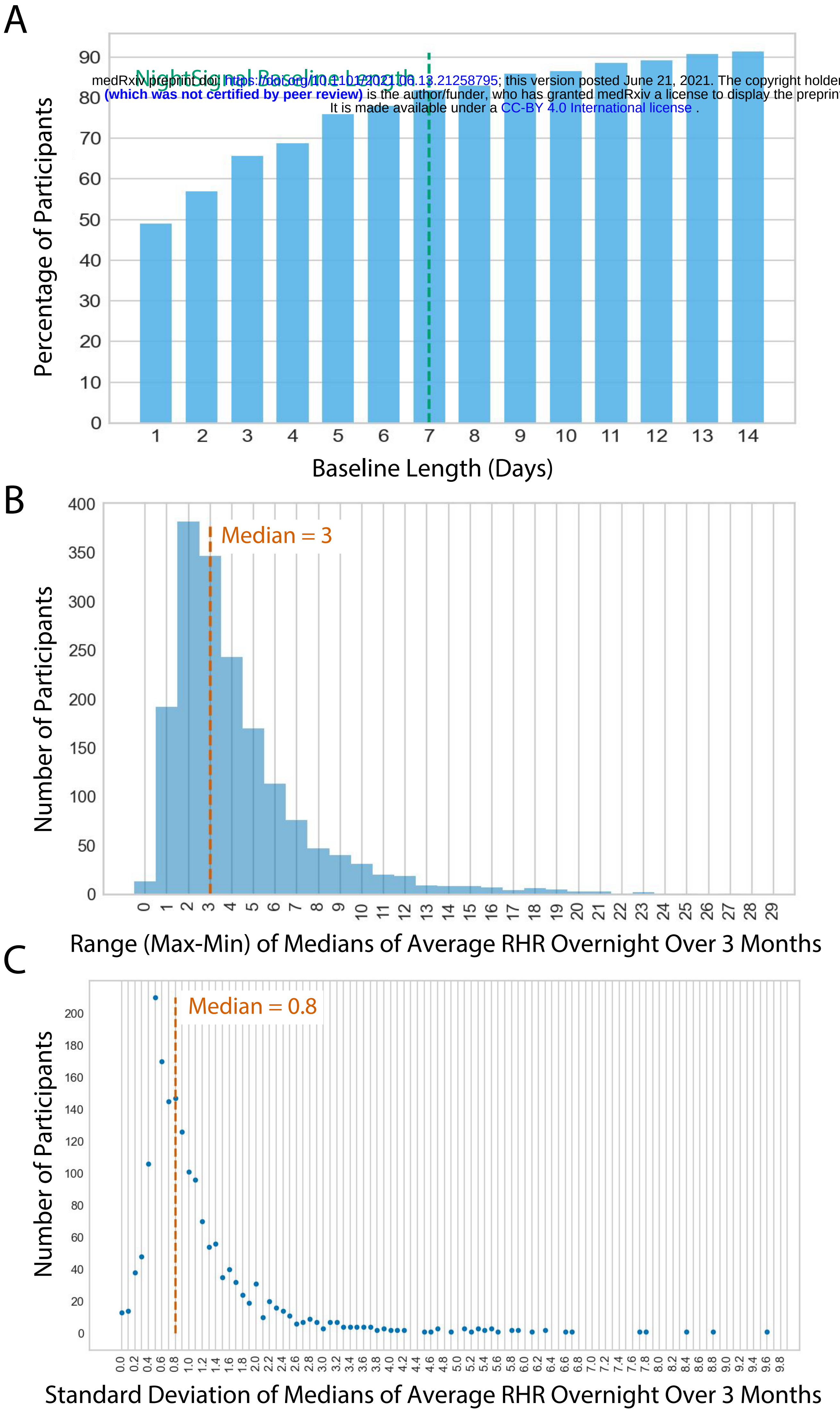

Figure S5: Examples of COVID-19 Positive and Negative, and Potentially Healthy Cases

medRxiv preprint doi: https://doi.org/10.1101/2021.06.13.21258795; this version posted June 21, 2021. The copyright holder for this preprint (which was not certified by peer review) is the author/funder, who has granted medRxiv a license to display the preprint in perpetuity.
It is made available under a CC-BY 4.0 International license .

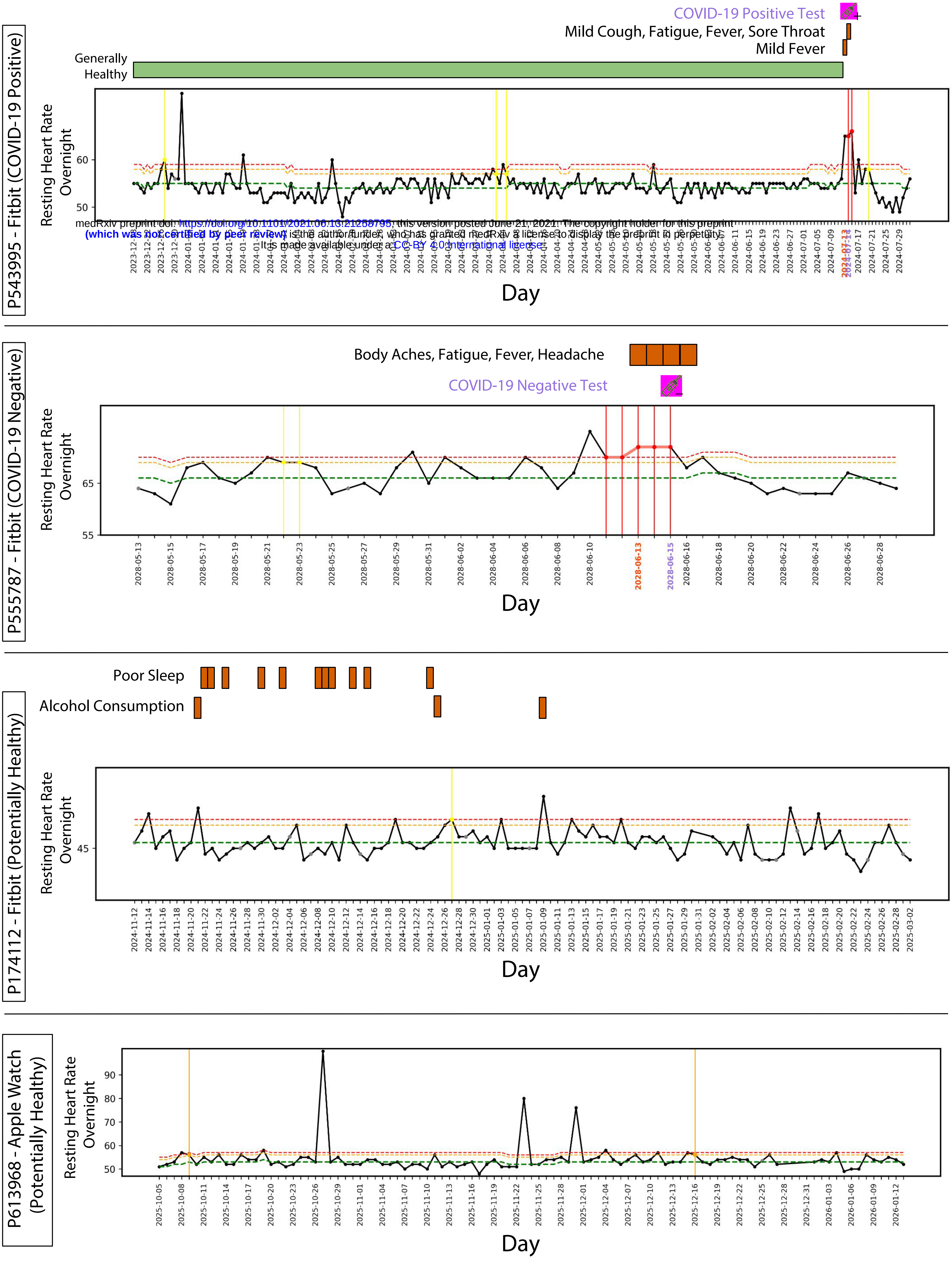

Figure S6: Impact of Different Events on Signals (All Day vs. Overnight)

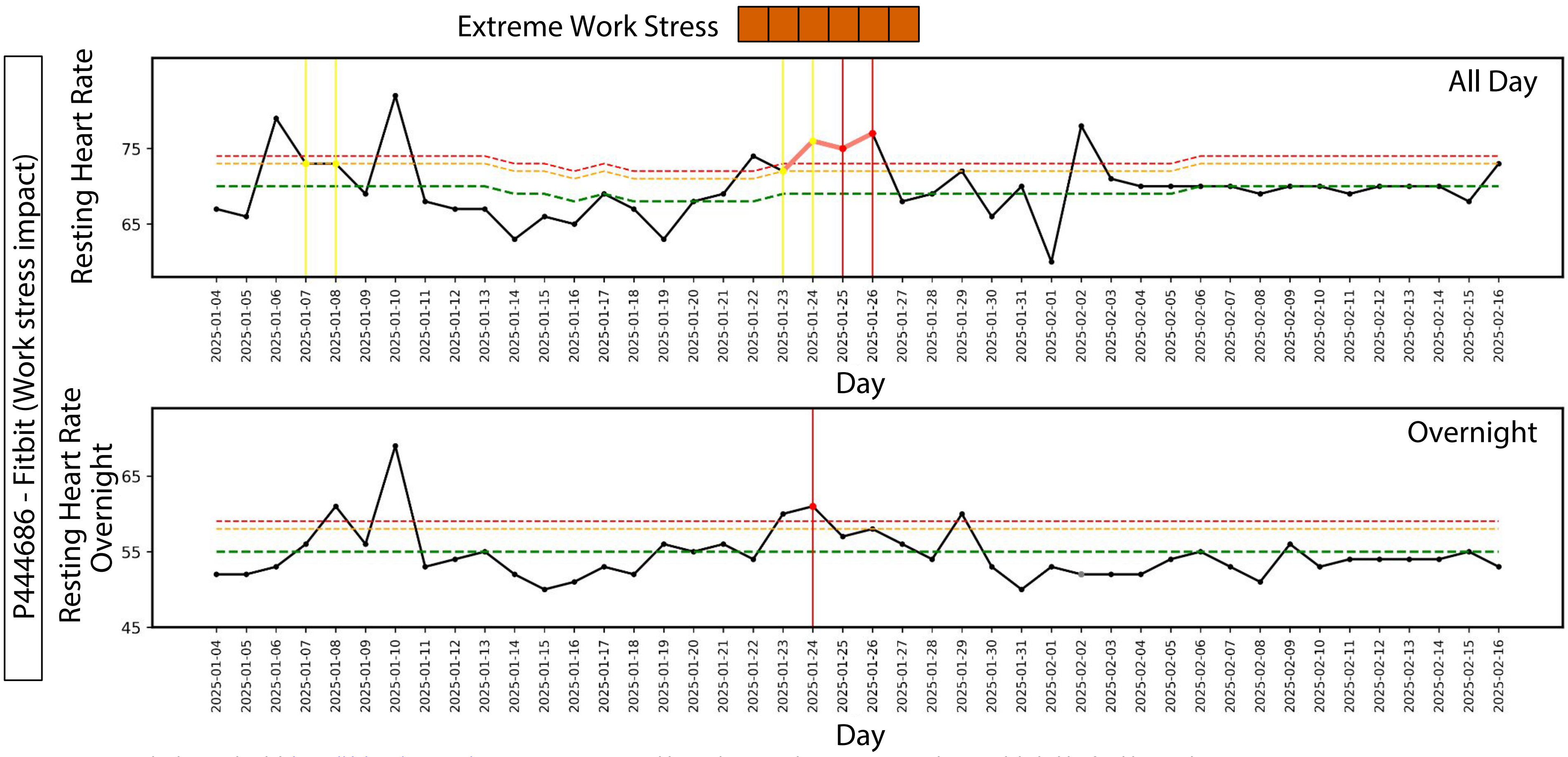

medRxiv preprint doi: https://doi.org/10.1101/2021.06.13.21258795; this version posted June 21, 2021. The copyright holder for this preprint (which was not certified by peer review) is the author/funder, who has granted medRxiv a license to display the preprint in perpetuity.
It is made available under a CC-BY 4.0 International license .

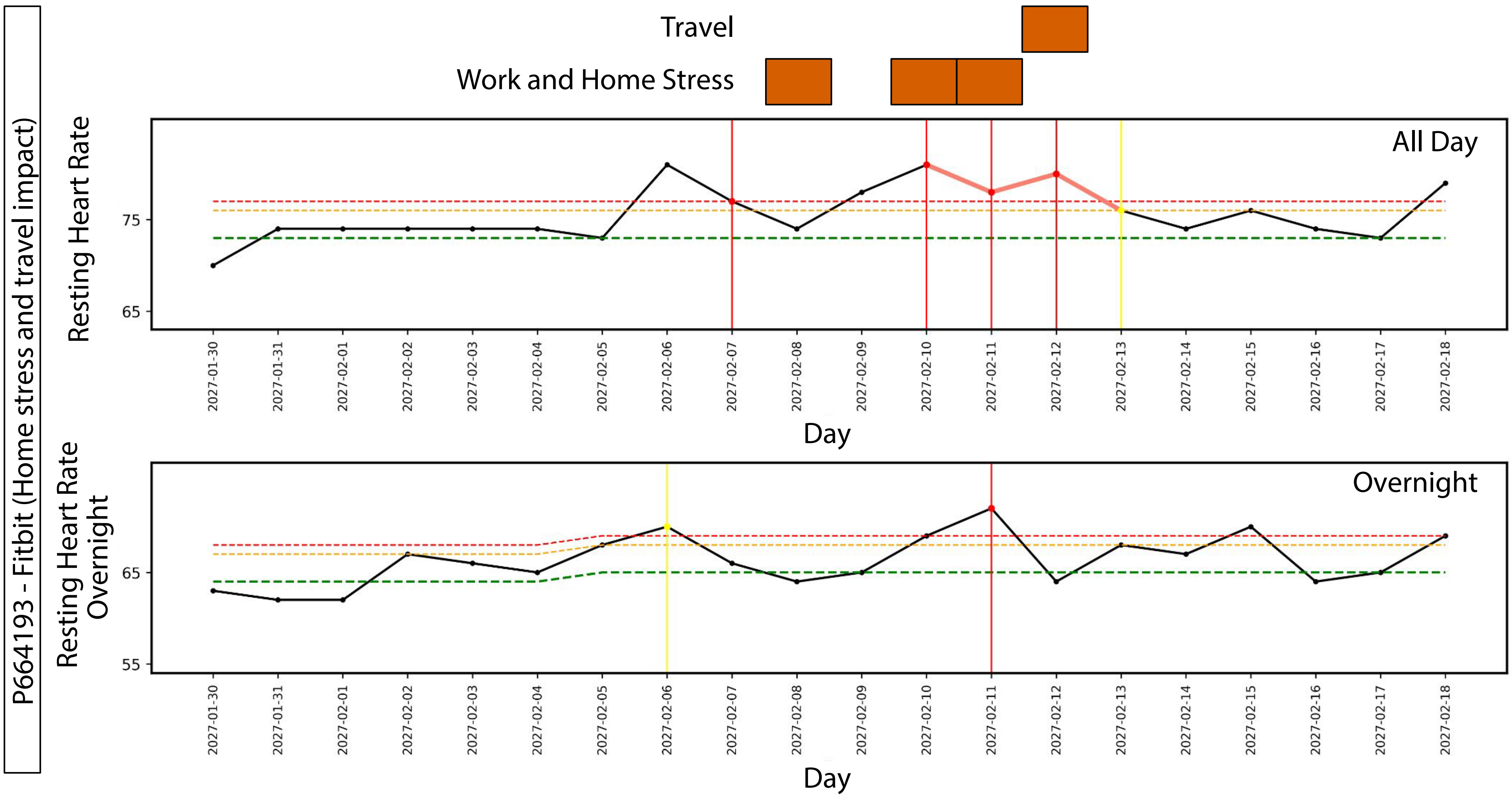

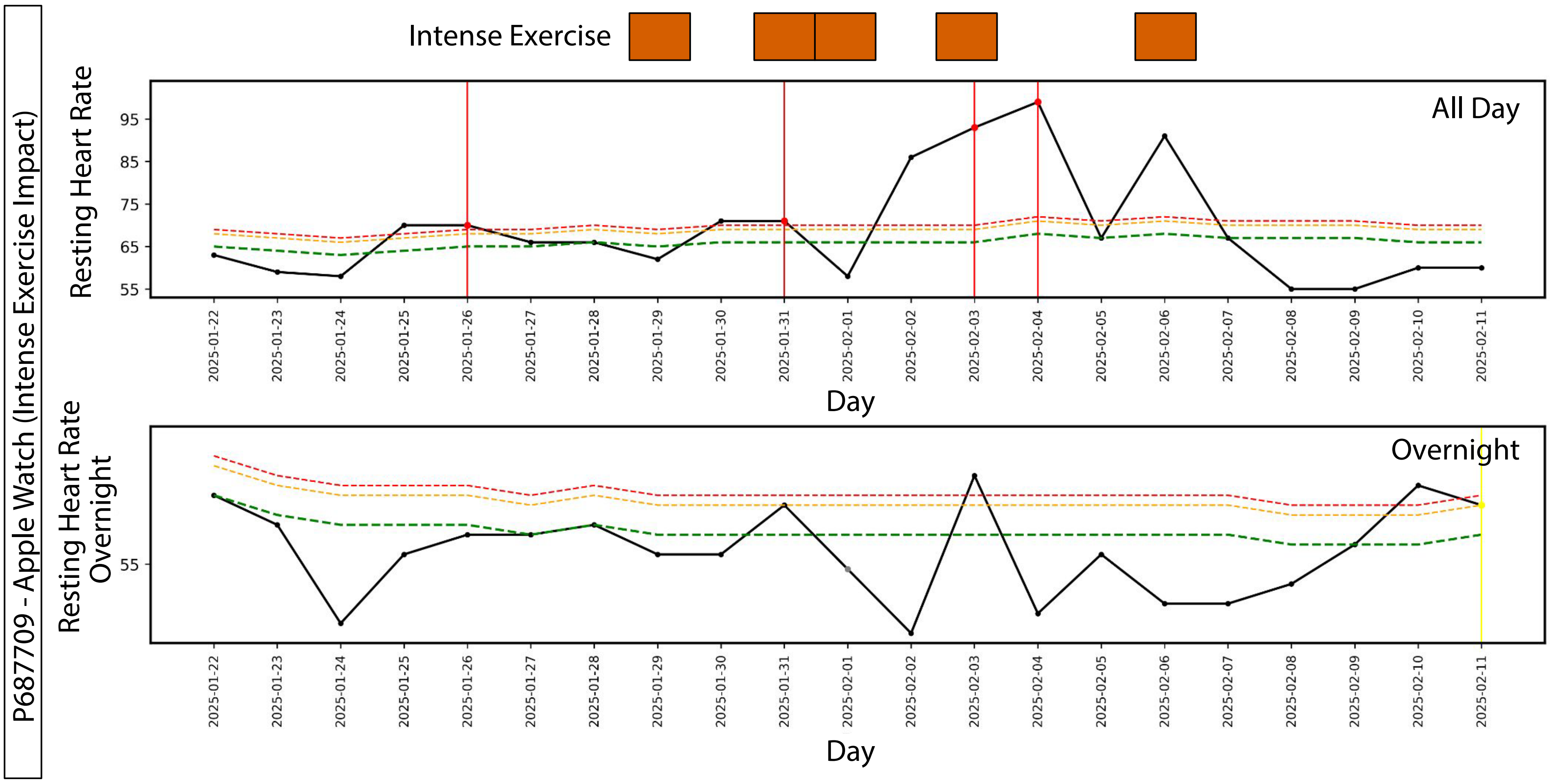

Figure S7: Winter Holidays Bumps

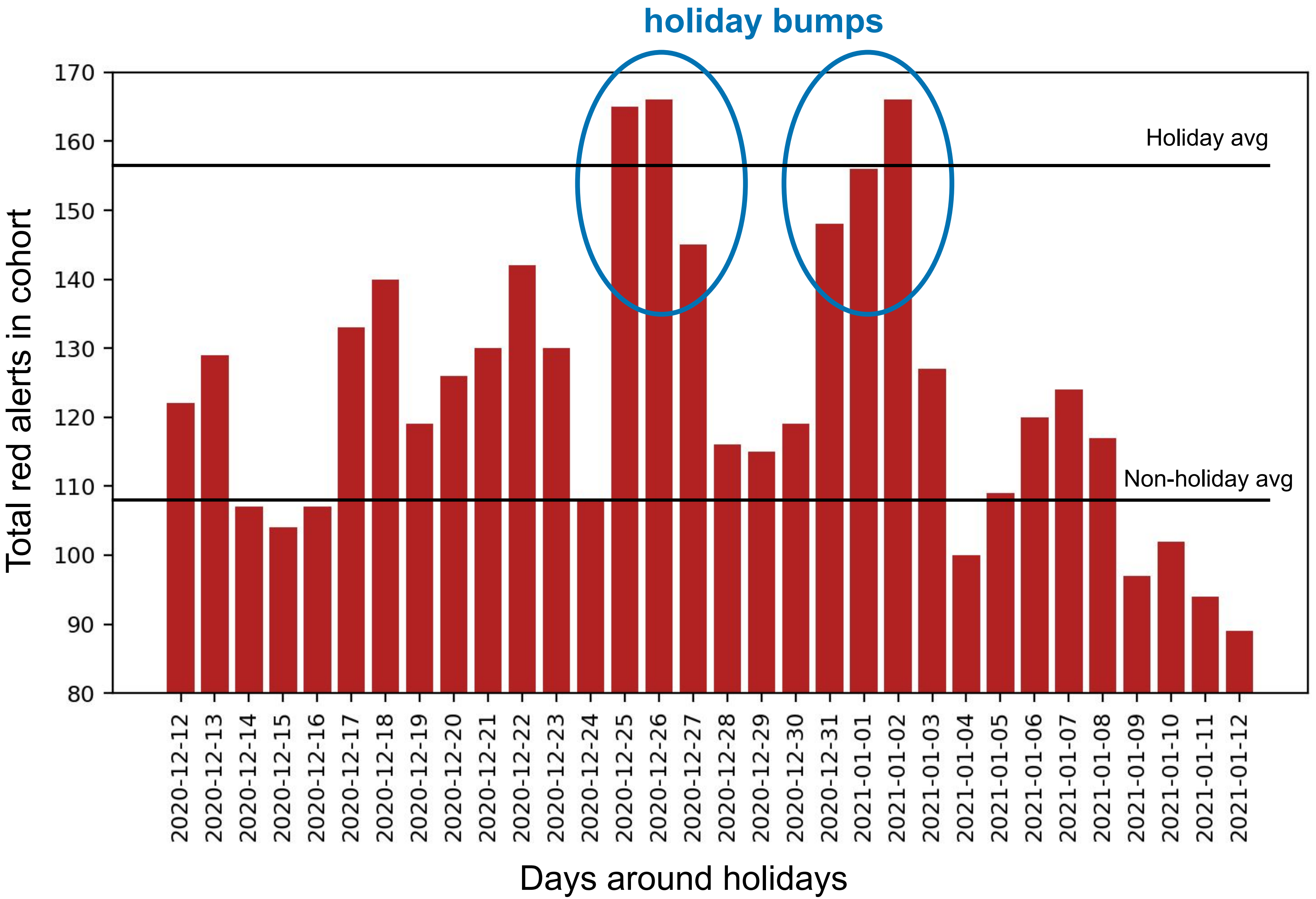

Supplement: 1 [file NIHPP2021.06.13.21258795V1-supplement-1.pdf]
